# ecGBMsub: an integrative stacking ensemble model framework based on eccDNA molecular profiling for improving IDH wild-type glioblastoma molecular subtype classification

**DOI:** 10.3389/fphar.2024.1375112

**Published:** 2024-04-11

**Authors:** Zesheng Li, Cheng Wei, Zhenyu Zhang, Lei Han

**Affiliations:** ^1^ Tianjin Neurological Institute, Key Laboratory of Post-Neuro Injury, Neuro-Repair and Regeneration in Central Nervous System, Ministry of Education and Tianjin City, Tianjin Medical University General Hospital, Tianjin, China; ^2^ Department of Neurosurgery, The First Affiliated Hospital of Zhengzhou University, Zhengzhou, Henan, China

**Keywords:** brain tumor, IDH wild-type glioblastoma, eccDNA, machine learning, ensemble model, molecular subtype

## Abstract

IDH wild-type glioblastoma (GBM) intrinsic subtypes have been linked to different molecular landscapes and outcomes. Accurate prediction of molecular subtypes of GBM is very important to guide clinical diagnosis and treatment. Leveraging machine learning technology to improve the subtype classification was considered a robust strategy. Several single machine learning models have been developed to predict survival or stratify patients. An ensemble learning strategy combines several basic learners to boost model performance. However, it still lacked a robust stacking ensemble learning model with high accuracy in clinical practice. Here, we developed a novel integrative stacking ensemble model framework (ecGBMsub) for improving IDH wild-type GBM molecular subtype classification. In the framework, nine single models with the best hyperparameters were fitted based on extrachromosomal circular DNA (eccDNA) molecular profiling. Then, the top five optimal single models were selected as base models. By randomly combining the five optimal base models, 26 different combinations were finally generated. Nine different meta-models with the best hyperparameters were fitted based on the prediction results of 26 different combinations, resulting in 234 different stacked ensemble models. All models in ecGBMsub were comprehensively evaluated and compared. Finally, the stacking ensemble model named “XGBoost.Enet-stacking-Enet” was chosen as the optimal model in the ecGBMsub framework. A user-friendly web tool was developed to facilitate accessibility to the XGBoost.Enet-stacking-Enet models (https://lizesheng20190820.shinyapps.io/ecGBMsub/).

## 1 Introduction

Glioblastoma (GBM) is a type of notorious intracranial tumor with low survival rates and high heterogeneity and recurrence rates. The standard treatment strategy involves surgery, radiation, and chemotherapy. While standard treatment can effectively alleviate symptoms, the overall survival of patients remains unsatisfactory (Lu et al., 2022; Schaff and Mellinghoff, 2023). In past decades, high-throughput omics data revealed heterogeneous genetic/genomic/epigenetic landscapes and prompted individual therapies based on molecular subtypes. In 2010, Verhaak et al. identified four GBM subtypes, including classical (CL), mesenchymal (MES), neural (NE), and proneural (PN) (Verhaak et al., 2010). Because PN contributes to a more favorable outcome while the MES subtype relates to dismal survival, numerous studies have focused on them (Yan et al., 2022; Qiu et al., 2023). However, the above-mentioned finding might be largely attributed to the favorable outcome of IDH mutant GBMs, which are consistently classified as PN. According to the fifth edition of the WHO Classification of Tumors of the Central Nervous System (WHO CNS5), the definition of GBM was augmented with genetic modifiers (e.g., GBM and IDH-wildtype) (Louis et al., 2021). In 2017, Wang et al. defined three IDH wild-type GBM subtypes based on the gene expression profile, including CL, MES, and PN, which are tightly related to the tumor immune environment (Wang et al., 2017). Their findings might aid in precision immunotherapy medicine. However, two potential issues need to be further improved. First, most clinicians are not familiar with the analysis of sequence data, which prevents them from calculating the patient’s molecular subtype based on gene expression information. Second, extrachromosomal genetic elements play a key role in regulating gene expression, but their roles in the identification of molecular subtypes have not been fully considered.

Extrachromosomal circular DNA (eccDNA) plays diverse roles in healthy bioprocesses and cancer progression. EccDNAs are mostly shorter than 1 kb. In contrast, a special subtype of eccDNA is named extrachromosomal DNA (ecDNA) and is much larger (50kb–5 Mb) (Yi et al., 2022). Although ecDNAs play an important role in cancer, the larger size makes it challenging for enrichment and identification, setting up a barrier for research. Compared with ecDNAs, eccDNAs with smaller sizes exhibit more promise serving as biomarkers. A previous study reported that eccDNAs in maternal plasma represent prospective circulating nucleic acid biomarkers to improve early diagnosis and management (Sin et al., 2020; Sin et al., 2021). Here, we adapt the Circle-seq to specifically enrich extrachromosomal circular DNA from GBM tumor tissues and adjacent non-tumor tissues. Circle-seq can effectively capture eccDNAs, although it is challenging to capture megabase-sized ecDNAs.

Leveraging machine learning to decode the underlying links in multi-omics biological data has been popular (Sin et al., 2021; Greener et al., 2022). Machine learning is a robust tool for tackling challenging data (Greener et al., 2022). Stacking ensemble is a strategy that combines the predictions of multiple base models to create an ensemble model with more robust performance. Here, we developed “ecGBMsub,” a stacking ensemble framework based on eccDNA molecular profiling, which may provide a convenient tool for clinicians to identify the IDH wild-type GBM subtypes and reveal the underlying links between GBM subtype evaluation and eccDNA biology.

We used nine machine learning algorithms to fit base models on the training set. Then, the five optimal base models on cross-validation (CV) were selected to create the stacking ensemble framework, ecGBMsub. The optimal stacking ensemble model, named XGBoost.Enet-stacking-Enet, exhibited robust performance in the prediction of all IDH wild-type GBM molecular subtypes. We conducted a model interpretability analysis using SHAP to understand the decision-making process. Finally, we developed a web tool and deployed the machine learning model on the web tool. Our studies might help clinicians predict molecular subtypes in patients with IDH wild-type GBM.

## 2 Materials and methods

### 2.1 Sample acquisition and diagnosis

GBM tumors and adjacent tissues (three pairs) were collected from First Affiliated Hospital of Zhengzhou University. Following WHO CNS5 standards, the initial histopathological diagnosis is conducted at the institution that collects the tissue. To ensure consistency across samples, in-house neuropathologists reviewed the initial diagnosis. This study was approved by the hospital’s institutional review board, and written informed consent was obtained from all patients. The clinical data for the samples are listed in Supplementary Table S1.

### 2.2 Circle-seq and data analysis

Circle-seq analysis based on clinical samples was performed by CloudSeq Biotech Inc. (Shanghai, China). The detailed procedure was previously reported (Møller et al., 2015; Møller et al., 2018). The edgeR (v0.6.9) software was used to normalize and calculate fold changes and *p*-values between two groups/samples to screen differentially expressed eccDNAs. Gene annotation of differential eccDNAs was performed using bedtools software (v2.27.1).

### 2.3 Data collection criteria and preprocess

The inclusion criteria for datasets were 1) that they contained IDH wild-type GBM and 2) that they contained classical, mesenchymal, and proneural molecular subtype information. Three datasets met the criteria and were enrolled in this study. The number of IDH wild-type GBM samples in these cohorts was as follows: TCGA-GBM (n = 114), CGGA301 (n = 89), and G-SAM (n = 283). The TCGA-GBM RNA-seq raw count was downloaded from the TCGA portal (https://portal.gdc.cancer.gov/) and converted to transcripts per kilobase million (TPM), followed by log-2 transformation. The G-SAM dataset was accessed from the European Genome Phenome Archive (EGA; EGAD00001007860) (Draaisma et al., 2020; Hoogstrate et al., 2023). The CGGA301 dataset was accessed from the Chinese Glioma Genome Atlas (CGGA, http://www.cgga.org.cn/) (Zhao et al., 2021). The ComBat algorithm was utilized to remove batch effects from nonbiological technical biases between different cohorts (Supplementary Figure S1). Somatic mutation and copy number segment data were downloaded from cBioPortal (https://www.cbioportal.org/) and FireBrowse (http://firebrowse.org/), respectively. TCGA was set as the training set, while the others (CGGA301 and G-SAM) were used for independent test datasets.

### 2.4 Molecular landscape analysis

The immune signatures were obtained from previous studies (Lu et al., 2021). The R package “GSVA” was used to calculate the score of immune signature gene sets in each sample. The R package “RTN” was used to construct transcriptional regulatory networks (regulons). The activities of regulons (transcription factors) that were associated with GBM progression and the regulators that were relevant to cancerous histone modification were calculated. Specifically, the transcriptome expression profile was analyzed using mutual information analysis and Spearman rank-order correlation to determine the potential associations between regulators and potential targets. Permutation analysis was applied to eliminate associations with an FDR >0.00001. Unstable associations were removed through a bootstrapping strategy of 1,000 resamplings with a consensus bootstrap greater than 95%. The weakest associations in the triangles of two regulators and common targets were removed. A two-sided GSEA was used to estimate the activity of each regulon.

### 2.5 Framework of ecGBMsub

**FIGURE 1:**
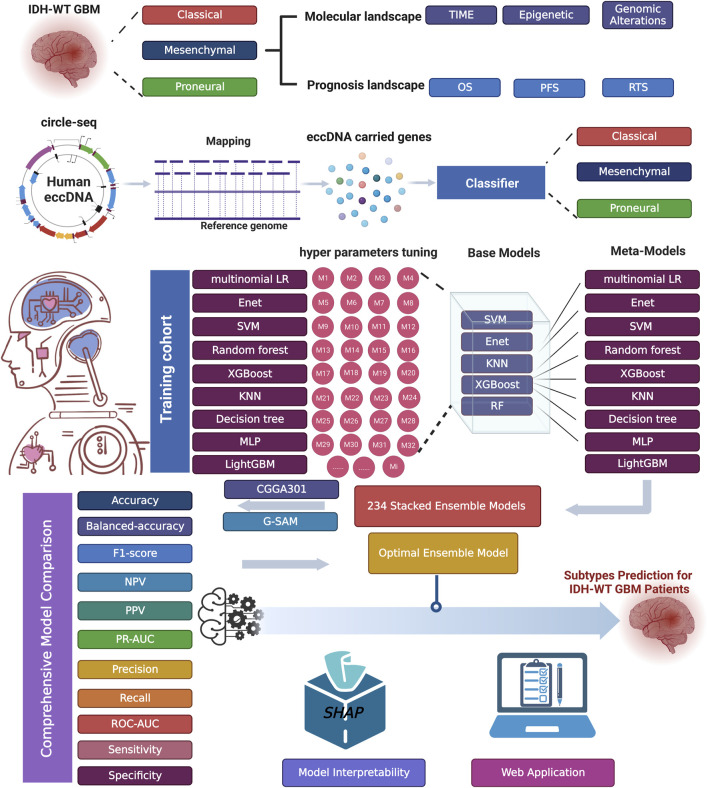
The workflow of this study.

Numerous studies have demonstrated that ensemble models might exhibit higher performance under certain situations than single models. Unlike other ensemble strategies, such as bagging and boosting, a stacking strategy creates a hybrid model by combining the strengths of different predictive models. The workflow of ecGBMsub development includes the fit, evaluation, and selection of base models, the generation of new feature vectors, the selection of meta-models with an enumeration method, and the selection of the optimal model by comprehensive evaluation. The workflow of this study is shown in Figure 1.

#### 2.5.1 Base model construction

Nine machine learning algorithms, including multinomial logistic regression, decision tree (DT), random forest (RF), extreme gradient boosting (XGBoost), multilayer perceptron (MLP), elastic net (Enet), support vector machine (SVM), light gradient boosting machine (LightGBM) and K-nearest neighbor (KNN), were performed to fit models based on 5-fold CV on the training cohort.

#### 2.5.2 Evaluation and selection of base models

Base learner accuracy and diversity were vital to the performance of the ensemble model. A comprehensive evaluation involving multiple metrics was performed. We used accuracy, balanced accuracy, F1-score, NPV, PPV, PR-AUC, precision, recall, ROC-AUC, sensitivity, and specificity in CV as statistics to ensure the robustness of base models. Finally, SVM, Enet, KNN, XGBoost, and RF were determined to be the best-performing base models.

#### 2.5.3 New feature vector generation

The new feature vector was created using predicted probabilities (PPs) of five optimal base models. The new feature vector was defined as [PPs(SVM), PPs(Enet), PPs(KNN), PPs(XGBoost), PPs(RF)]^T^, where PPs i) is the PPs based on the optimal base model i.

#### 2.5.4 Meta-model selection with enumeration method

Nine machine learning algorithms, including multinomial logistic regression, DT, RF, XGBoost, MLP, Enet, SVM, LightGBM, and KNN, were used as meta-models. In the enumeration framework, the five base models were randomly combined to generate 26 different combinations, and then nine algorithms were used to build a meta-model, resulting in 234 stacking models. In total, 243 types of models, including 234 stacking ensemble models and nine base models, were created in the ecGBMsub framework.

#### 2.5.5 Selection of optimal model by comprehensive evaluation

The performance of all models in ecGBMsub was evaluated and compared using 11 standard performance metrics for the classification problem: accuracy, balanced accuracy, F1-score, NPV, PPV, precision, recall, PR-AUC, ROC-AUC, sensitivity, and specificity. Finally, the stacking model, named XGBoost.Enet-stacking-Enet, was chosen as the optimal model in the ecGBMsub framework. XGBoost.Enet-stacking-Enet refers to a two-layer stacking model, of which the optimal XGBoost and Enet models are the base learner, and the Enet model is the meta-learner.

#### 2.5.6 Hyperparameter settings

The base model employs 5-fold cross-validation along with Bayesian optimization to improve its performance. The stacking ensemble model leverages bootstrap resampling in combination with Bayesian optimization to enhance performance. The hyperparameter tuning metrics were set as balanced accuracy. During Bayesian optimization, the maximum number of iterations is set to 50. If there is no performance improvement within 10 consecutive iterations, the process is terminated early. The bootstrap resampling times are set to 10. The hyperparameter tuning process is shown in Supplementary Figure S2.

### 2.6 Statistical analysis

The statistical tests were conducted by R4.2.2, including the Kruskal–Wallis test for comparisons among multiple groups of continuous variables, Fisher’s exact test for categorical data, and the log-rank test for Kaplan–Meier curves. For unadjusted comparisons, statistical significance was defined as *p* < 0.05.

## 3 Results

### 3.1 Prognostic landscape of IDH wild-type GBM subtypes

**FIGURE 2:**
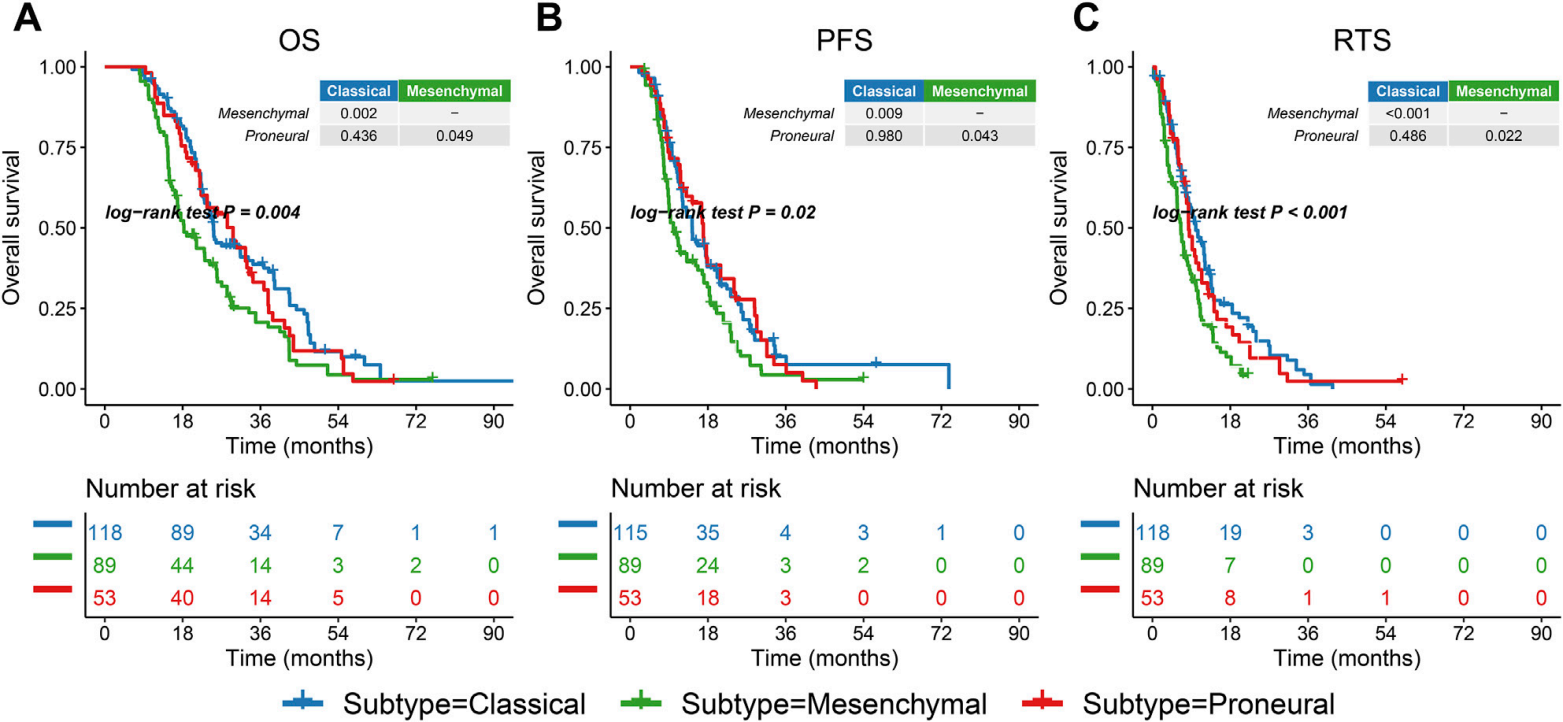
Prognostic landscape of three IDH wild-type GBM subtypes. **(A–C)** Kaplan–Meier curves of overall survival (OS), progression-free survival (PFS), and post-relapse treatment survival (RTS) log-rank test for 260 IDH wild-type GBM patients classified by subtypes.

Most of the patients in the G-SAM database received standard treatment, while the treatment approaches of patients in the TCGA group were diverse. Therefore, the G-SAM database was selected for the survival analysis. To standardize the survival analysis candidates, the patients were filtered according to the following criteria: 1) IDH wild-type; 2) underwent temozolomide chemotherapy combined with radiotherapy after surgical resection. Patients meeting both criteria were retained for survival analysis (n = 260). The mesenchymal subtype in the G-SAM cohort had the most unfavorable overall survival (OS), progression-free survival (PFS), and post-relapse treatment survival (RTS) of all the subtypes (all pairwise comparisons, *p* = 0.004; *p* = 0.02; *p* < 0.001, Figures 2A–C). RTS refers to the survival time after the patient’s tumor recurred and received treatment. Thus, different subtypes imply different prognostic landscapes, which emphasizes the importance of accurate prediction of subtypes.

### 3.2 Molecular landscape of IDH wild-type GBM subtypes

**FIGURE 3:**
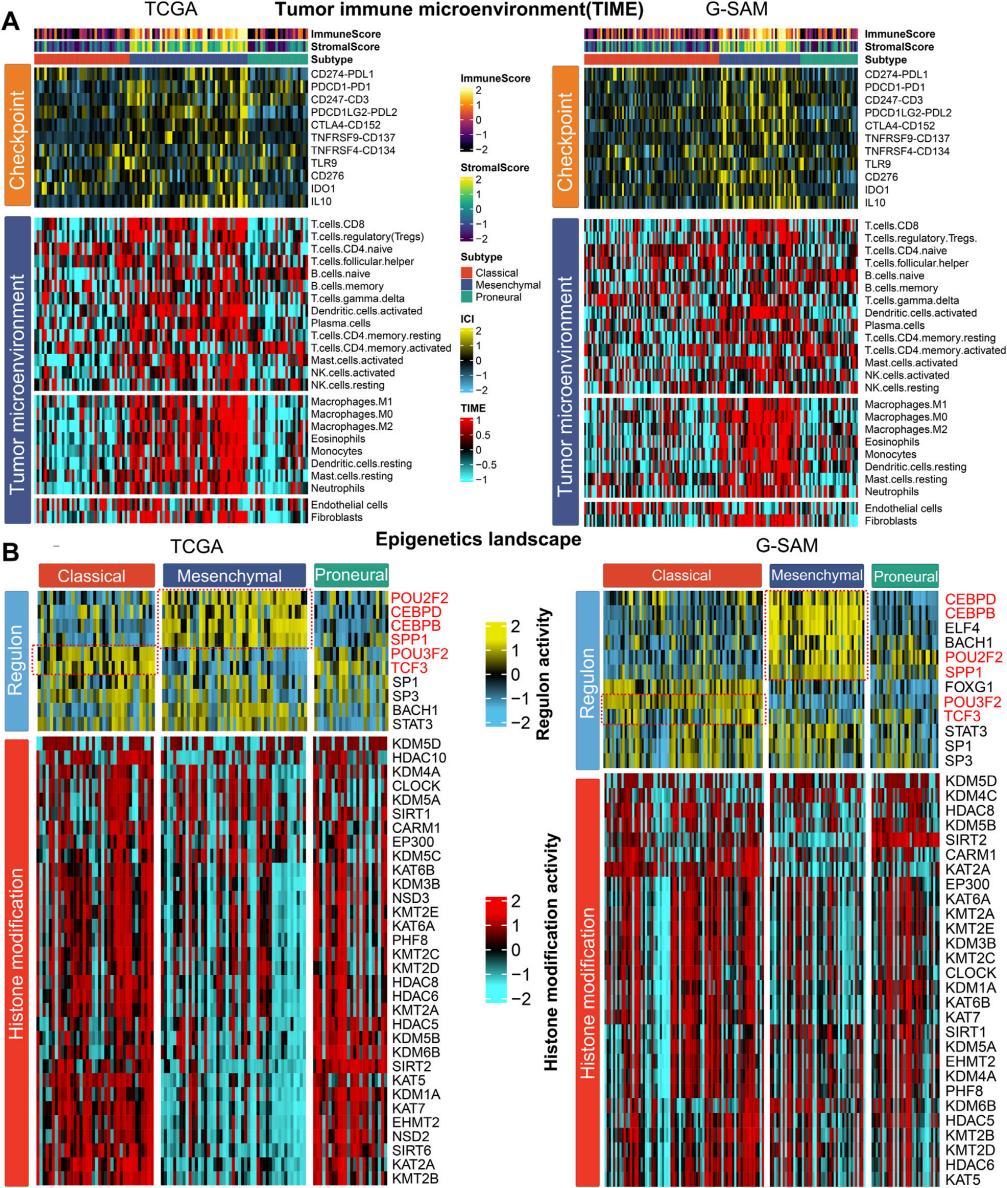
Molecular landscape of three IDH wild-type GBM subtypes. **(A)** Tumor immune microenvironment (TIME): heatmap showing the immune profile in the IDH wild-type GBM subtypes (TCGA for left; G-SAM for right), with the top panel showing the expression of genes involved in immune checkpoint targets and the bottom panel showing the enrichment level of 24 microenvironment cell types. The immune enrichment score and stromal enrichment score are annotated at the top of the heatmap. **(B)** Epigenetic landscape: heatmap showing regulon activity profiles for GBM transcription factors (top panel) and potential regulators associated with histone modification (bottom panel) in the IDH wild-type GBM subtypes (TCGA on the left; G-SAM on the right).

As cancer immunity plays a pivotal role in tumor progression, we hypothesized that the tumor immune microenvironment (TIME) of the mesenchymal subtype may be different from that of the other subtypes. Thus, the immune cell infiltration status was investigated in two cohorts (TCGA and G-SAM). Specifically, we quantified the infiltration levels of 24 microenvironment cell types and the expression of immune checkpoints in IDH wild-type GBM samples. In the TCGA and G-SAM cohorts, the analysis of immune signature expression levels suggested that immunocyte infiltration was dramatically higher in the mesenchymal subtype (Figure 3A; Supplementary Figure S3A). Compared to the other subtypes, the mesenchymal subtype exhibited a relatively higher expression of checkpoints that represent potential targets for immunotherapy (Lu et al., 2021), including *CD247* (*CD3*), *CTLA4* (*CD152*), *IDO1*, *IL10*, *PDCD1*, *PDCD1LG2* (*PDL2*), and *TNFRSF9* (*CD137*) in the TCGA cohort and *CD247* (*CD3*), *CD274* (*PDL1*), *CD276*, *CTLA4* (*CD152*), *IDO1*, *IL10*, *PDCD1* (*PD1*), *PDCD1LG2* (*PDL2*), and *TNFRSF9* (*CD137*) in the G-SAM cohort (Supplementary Figure S3B). Additionally, the mesenchymal subtype exhibited higher immune and stromal scores (Figure 3A; Supplementary Figure S3C), which confirms the feasibility of microenvironment-targeted therapy for patients with the mesenchymal subtype.

To further explore the epigenetic landscape of IDH wild-type GBM, we analyzed GBM-specific regulon activity and potential regulators relevant to cancerous histone modification (Li et al., 2016; Sharpe and Baskin, 2016; Luedi et al., 2018; Chen et al., 2019; Yang et al., 2021; Tsai et al., 2022; Yuan et al., 2022; Dai et al., 2023; Kosti et al., 2023; Mao et al., 2023; Yao and Wang, 2023). Surprisingly, the regulon activity was tightly associated with IDH wild-type GBM subtypes. The patterns of regulon activity were different in three subtypes. The mesenchymal subtype exhibits high activity of *POU2F2*, *SPP1*, *CEBPD*, and *CEBPB*, while the classical subtype was distinctly associated with high activity of *POU3F2* and *TCF3* (Figure 3B). The regulation activity patterns associated with histone modification among the three subtypes were also different, emphasizing that epigenetic networks might play a pivotal role in defining these molecular subtypes. Compared with classical and proneural subtypes, the mesenchymal subtype exhibited lower cancerous histone modification activity in the TCGA cohort (Figure 3B). Therefore, epigenetic regulation of the mesenchymal subtype may be primarily influenced by transcription factor activity rather than chromatin accessibility.

**FIGURE 4:**
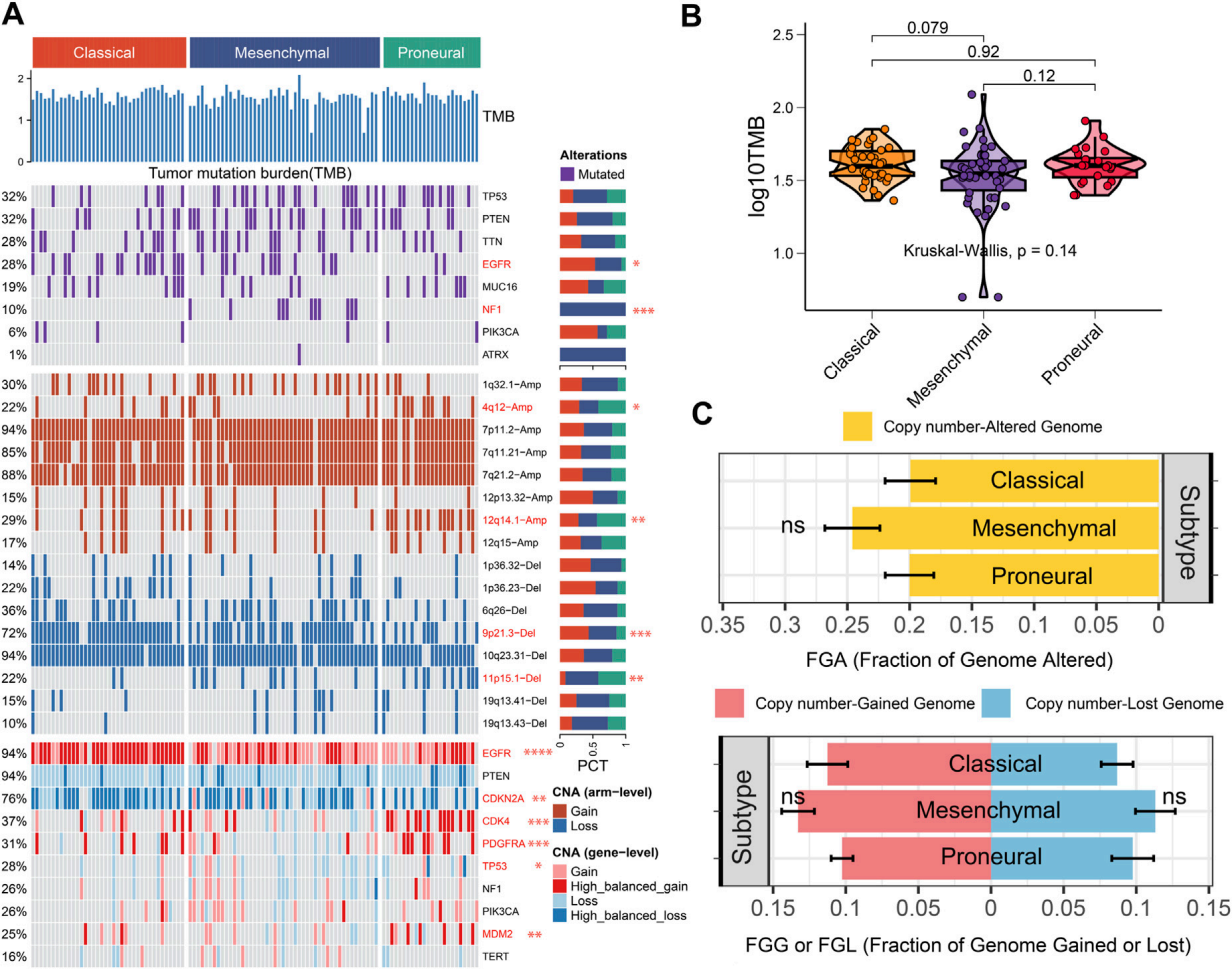
Genomic alteration landscape of three IDH wild-type GBM subtypes. **(A)** Genomic alteration landscape in the three IDH wild-type GBM subtypes. **(B)** Tumor mutation burden (TMB) in different subtypes. **(C)** Distribution of fraction genome altered (FGA) and fraction genome gain/loss (FGG/FGL) in the three IDH wild-type GBM subtypes. Bar charts are presented as the mean ± standard error of the mean. * (*p* < 0.05), ** (*p* < 0.01), and *** (*p* < 0.001), ns (*p* > 0.05).

To investigate the genomic heterogeneity of three IDH wild-type GBM subtypes further, the genomic alteration landscape was systematically characterized in the TCGA cohort (Figure 4A). There was no significant difference in tumor mutation burden (TMB) among subtypes (*p* = 0.14, Figure 4B). The fraction genome altered (FGA) and fraction genome gain/loss (FGG/FGL) among the three subtypes did not show differences (Figure 4C).

Notably, we found that the classical and mesenchymal subtypes exhibited higher *EGFR* mutation frequencies than the proneural subtype. The *NF1* was exclusively mutated in mesenchymal subtype (Figure 4A). Wang et al. reported that *NF1* deficiency drives the infiltration of tumor-associated macrophages/microglia (Wang et al., 2017). Therefore, *NF1* mutations may contribute to a tumor-promoting immune microenvironment in the mesenchymal subtype. Meanwhile, the proneural subtype exhibited higher 4q12-amplification, 12q14.1-amplification, and lower 9p21.3-deletion than other subtypes. The classical subtype exhibited lower 11p15.1-deletion than other subtypes. Alterations in these chromosomal arms may serve as biomarkers for subtype identification. Additionally, *EGFR* showed a more high-balanced gain in the classical subtype than other subtypes, which is consistent with previous studies (Verhaak et al., 2010). EGFR tyrosine kinase inhibitor (TKI) might exhibit better benefits for patients in the classical subtype. *CDKN2A* showed more high-balanced loss in the classical and mesenchymal subtypes than in the proneural subtype. Nathanson et al. reported that *CDKN2A* deletion remodels lipid metabolism, thereby priming GBM for ferroptosis (Minami et al., 2023). Drugs targeting lipid metabolism and enhancing the ferroptosis process may be more suitable to benefit patients with the classic or the mesenchymal subtypes. The *CDK4*, *PDGFRA*, and *MDM2* showed more high-balanced gain in the proneural than the other subtypes, which provided potential individualized targets for patients in the proneural subtype (Figure 4A). Meanwhile, some inhibitors that might aid in clinical administration have been developed, including CDK4/6 inhibitors, PDGFRA inhibitors, and MDM2 inhibitors. Therefore, considering genetic alterations in clinical administration may be the key to achieving precision medicine among different subtypes.

### 3.3 Feature selection and base model construction

A total of 924 differentially expressed eccDNAs carrying protein-coding genes were identified in GBM core tissues and adjacent tissues with a cut-off criteria of |FC (fold change)| >2 and *p* < 0.05. Among them, 371 eccDNAs were upregulated, and 553 were downregulated in the GBM tumor core tissues (Supplementary Table S2). Finally, 717 protein-coding genes carried by differentially expressed eccDNAs were included in three data sets (TCGA, CGGA301, and G-SAM). The 717 protein-coding genes mentioned above are original features and will be used for subsequent analysis.

**FIGURE 5:**
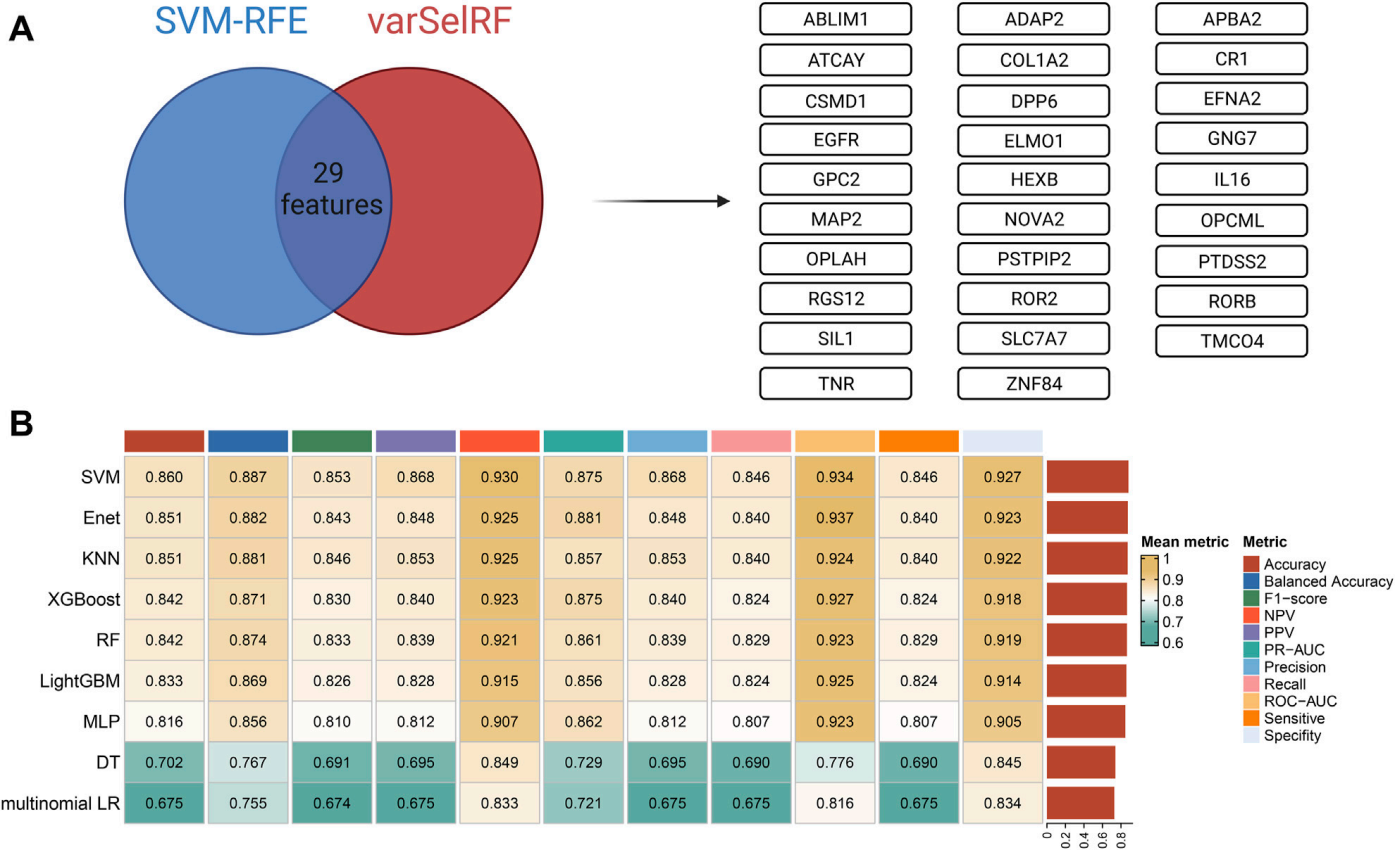
Feature selection and base model construction and comparison. **(A)** Feature selection: the SVM-REF and varSelRF algorithms were used for feature selection, and 29 features were retained. **(B)** Base model construction and comparison: nine machine learning algorithms were used to fit models based on 5-fold cross-validation (CV) on the training cohort (TCGA). The performance of each model on CV was comprehensively evaluated.

Two machine learning algorithms, including SVM-RFE and varSelRF, were used for feature selection. When faced with redundant features, SVM-RFE generally exhibits higher robustness because it considers the interactions between the features. The varSelRF algorithm is based on multiple decision trees with non-linear modeling capabilities. The rationality of these two methods is that they can provide feature screening at different levels and perspectives. Finally, a total of 29 independent features were obtained based on two algorithms, including *ABLIM1*, *ADAP2*, *APBA2*, *ATCAY*, *COL1A2*, *CR1*, *CSMD1*, *DPP6*, *EFNA2*, *EGFR*, *ELMO1*, *GNG7*, *GPC2*, *HEXB*, *IL16*, *MAP2*, *NOVA2*, *OPCML*, *OPLAH*, *PSTPIP2*, *PTDSS2*, *RGS12*, *ROR2*, *RORB*, *SIL1*, *SLC7A7*, *TMCO4*, *TNR,* and *ZNF84* (Figure 5A).

Nine machine learning algorithms, including multinomial logistic regression, DT, RF, XGBoost, MLP, Enet, SVM, LightGBM, and KNN, were used to fit base models on the training set (TCGA cohort). The optimal hyperparameters on the CV of each base model are shown in Supplementary Table S3. The nine models with optimal hyperparameters were comprehensively evaluated on CV. Eleven metrics were calculated for model evaluation, including accuracy, balanced accuracy, F1-score, NPV, PPV, PR-AUC, precision, recall, ROC-AUC, sensitivity, and specificity. Finally, the mean metric was calculated as the average of each metric on CV (Figure 5B). SVM, Enet, KNN, XGBoost, and RF were the top five optimal base models among these models.

### 3.4 Construction of stacking ensemble models and selection of optimal model by comprehensive evaluation

We randomly combined the five optimal base models, which generated 26 types of different combinations. Then, the new feature vector was created using the predicted probabilities (PPs) of 26 combinations. For example, for the combination of SVM. Enet. KNN. XGBoost. RF, the new feature vector was defined as [PPs(SVM), PPs(Enet), PPs(KNN), PPs(XGBoost), PPs(RF)]^T^, where PPs i) is the PPs based on the optimal base model i. Next, the meta-models were fitted based on the new feature vector. Here, nine types of machine learning algorithms, including multinomial logistic regression, DT, RF, XGBoost, MLP, Enet, SVM, LightGBM, and KNN, were set as meta-models to fit the two-layer stacking ensemble models. Finally, 234 stacking ensemble models were generated.

**FIGURE 6:**
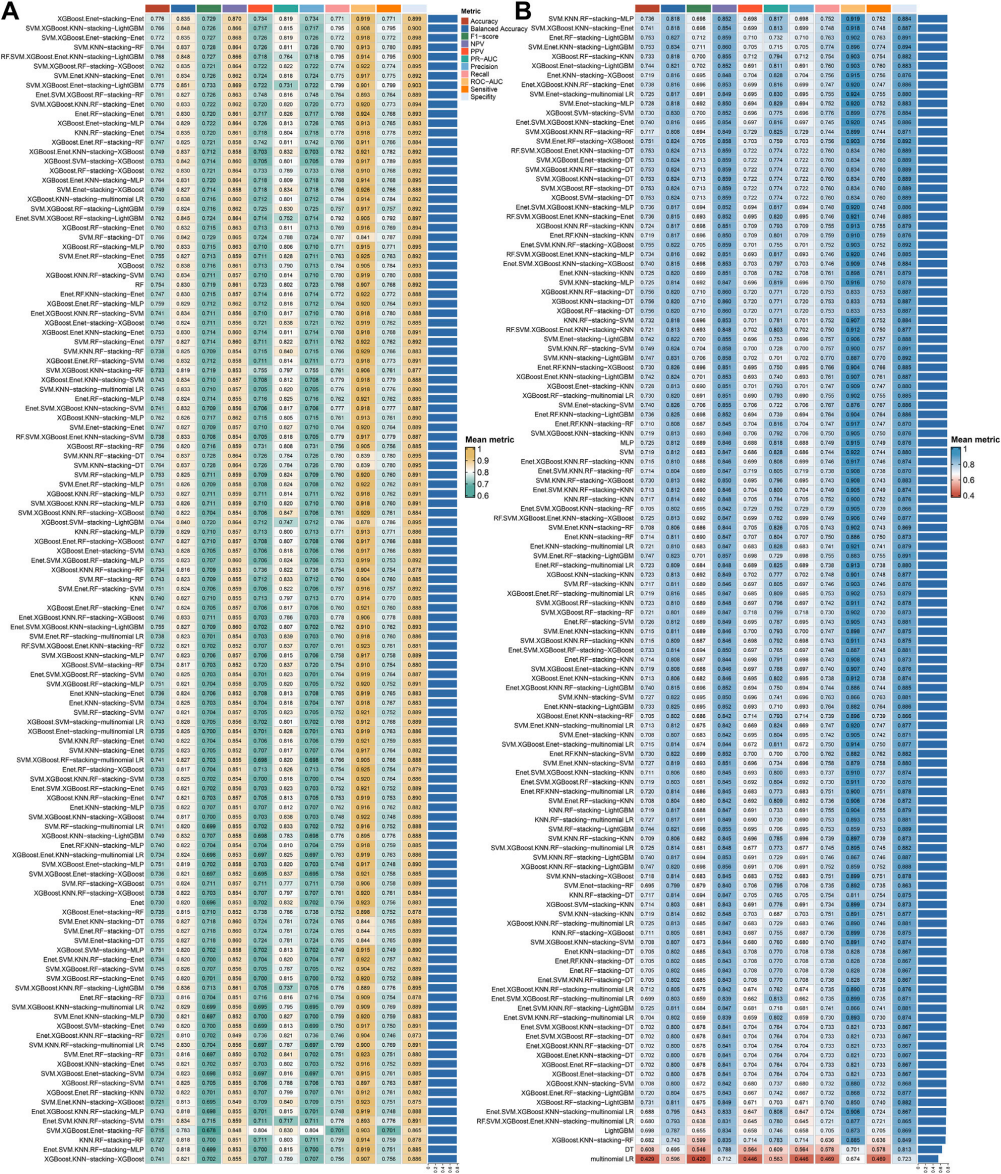
Integrative construction of stacking ensemble models and model comparison. A total of 243 models, including 234 stacking ensemble models and nine base models, were comprehensively evaluated on two test cohorts. The metric values were calculated by the mean values of the G-SAM and CGGA301 cohorts. **(A)** The top 121 models ranked by mean metric. **(B)** The 122–243 models ranked by mean metric.

The 243 models, including 234 stacking ensemble models and nine base models, were comprehensively evaluated on the G-SAM and CGGA301 test cohorts. The mean metric was calculated by the mean value of each metric on the two test sets. The XGBoost.Enet-stacking-Enet stacking ensemble model exhibits a robust performance among all metrics on test sets (Figure 6).

### 3.5 Evaluation of XGBoost.Enet-stacking-Enet

**FIGURE 7:**
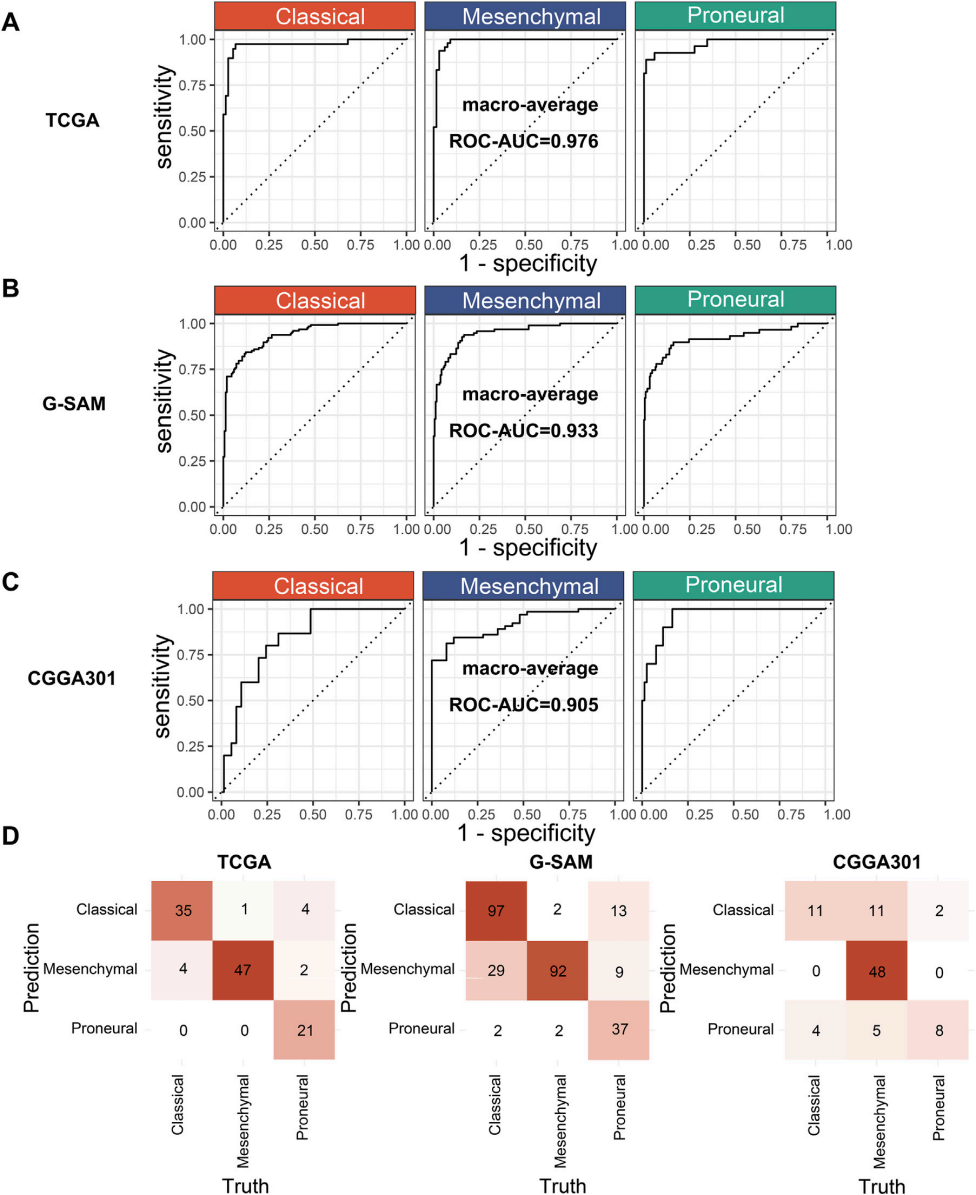
Performance evaluation of the optimal model. **(A)** ROC curves of the XGBoost.Enet-stacking-Enet model in the training cohort (TCGA). **(B, C)** ROC curves of the XGBoost.Enet-stacking-Enet model in the test cohort (G-SAM, CGGA301). **(D)** Confusion matrix of the XGBoost.Enet-stacking-Enet model on the training and test cohorts.

An ROC curve and a confusion matrix were used for the performance evaluation of XGBoost.Enet-stacking-Enet. ROC curves can exhibit the model’s classification ability across each class. The confusion matrix provides detailed class-level performance metrics. The macro average method was used to calculate the area under the curve (ROC-AUC) of the model on each class. This approach calculates and averages the AUC for each class to obtain an averaged ROC-AUC. In this way, we can evaluate the performance of each class equally without suffering from class imbalance. The macro-averaged ROC-AUC values were 0.976 in the TCGA cohort, 0.933 in the G-SAM cohort, and 0.905 in the CGGA301 cohort, respectively (Figures 7A–C). The confusion matrix consists of rows representing the true classes and columns representing the model’s predicted classes. Overall, the XGBoost.Enet-stacking-Enet model performed well on both the training and test sets (Figure 7D).

### 3.6 Interpretability analysis of XGBoost.Enet-stacking-Enet

**FIGURE 8:**
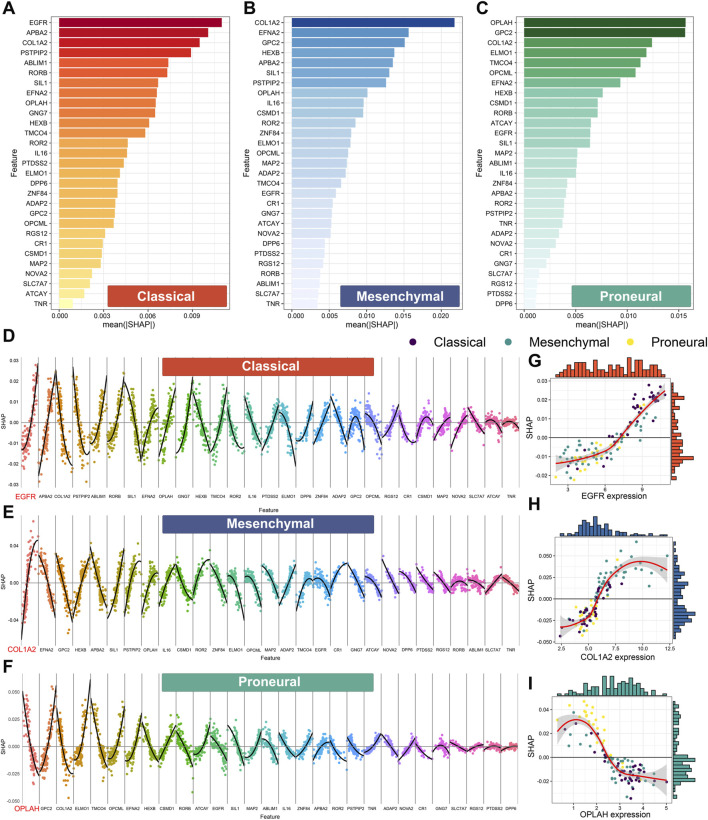
Feature importance based on Shapley additive explanation (SHAP) analysis of the XGBoost.Enet-stacking-Enet model. Feature importance for the prediction of the classical subtype **(A)**, the mesenchymal subtype **(B),** and the proneural subtype **(C)**. The features are ranked from most important to least important on the vertical axis, ordered from top to bottom. The horizontal axis shows the mean absolute SHAP values in all samples in the TCGA cohort. The higher absolute SHAP values mean higher importance. Feature contribution for the prediction of the classical subtype **(D)**, mesenchymal subtype **(E),** and proneural subtype **(F)**. The non-linear relationship between the expression level of EGFR **(G)**, COL1A2 **(H)**, OPLAH **(I),** and SHAP value. The horizontal axis represents the feature expression values of each feature ordered from low to high in each feature. SHAP values are presented on the vertical axis. A SHAP value higher than 0 implicates positive contributions to the corresponding subtype prediction, and SHAP values less than 0 indicate negative contributions to the corresponding subtype prediction.

Shapley additive explanations (SHAP) is widely used to explain machine learning model predictions. Here, SHAP was used to perform an interpretability analysis of XGBoost.Enet-stacking-Enet. First, the feature importance analysis was performed by calculating the mean absolute SHAP value. For the prediction of classical, mesenchymal, and proneural subtypes, the expression levels of *EGFR*, *COL1A2,* and *OPLAH/GPC2* play the most important roles (Figures 8A–C).

Next, we explored the contributional directions of features for each subtype. The high expression level of *EGFR* might contribute to the classical subtype (Figure 8D). Meanwhile, the high expression level of *COL1A2* and *GPC2* might contribute to mesenchymal and proneural subtypes, respectively (Figures 8E,F). Notably, the correlations between some features and prediction outcomes were non-linear, which confirmed the ability of XGBoost.Enet-stacking-Enet to capture non-linear relationships. For example, the expression levels of *EGFR*, *COL1A2,* and *OPLAH* have a non-linear relationship with outcomes (Figures 8G–I).

### 3.7 Establishment of web tool based on the XGBoost.Enet-stacking-Enet model

To support intelligent automated decision-making and make it easier for clinicians to use the model, we developed a web tool (https://lizesheng20190820.shinyapps.io/ecGBMsub/). This web tool will promote the practice of precision medicine for IDH wild-type GBM patients, ultimately improving patients’ quality of life and treatment outcomes.

## 4 Discussion

IDH wild-type GBM intrinsic subtypes have been linked to different genomic landscapes, epigenetic landscapes, treatment benefits, and tumor immune microenvironments (Figures 2–4). Accurately identifying molecular subtypes is pivotal to better understanding the biological characteristics of IDH wild-type GBM, thereby developing more personalized treatment administration. Our previous study demonstrated that eccDNA plays an important role in predicting the grade and prognosis of glioma patients and the recurrence of GBM (Li et al., 2023). Here, we would like to further explore the potential of eccDNA in the identification of IDH wild-type GBM molecular subtypes.

In this study, we have successfully identified differentially expressed eccDNAs between IDH wild-type GBM tumor tissues and para-tumor tissues through Circle-seq sequencing. Simultaneously, it was discovered that these differentially expressed eccDNAs carried several protein-coding genes. Currently available IDH wild-type GBM datasets with subtype information were collected. A novel stacked ensemble machine learning framework, ecGBMsub, was developed by integrating multiple machine learning algorithms. Following a comprehensive model evaluation and comparison, the XGBoost.Enet-stacking-Enet model was determined to be the optimal model (Figure 6). The XGBoost.Enet-stacking-Enet is a two-layer stacking ensemble model, utilizing the XGBoost and Enet models with optimal hyperparameters as base models and Enet as the meta-model. The meta-model takes the PPs of the base model as input and allows the generation of new PPs, thus integrating the advantages of different models. As expected, this model exhibits robust performance in predicting molecular subtypes in IDH wild-type GBM patients (Figure 7). To better understand how XGBoost.Enet-stacking-Enet achieves predictions for each subtype, a model interpretability analysis was conducted using SHAP to help explain the decision-making process for molecular subtype identification (Figure 8). To enhance the model’s clinical applicability, a website tool was developed, and the machine learning model was deployed on the website. This facilitates easy access for clinicians to leverage our model for molecular subtype prediction in IDH wild-type GBM patients.

Compared with previous models, the XGBoost.Enet-stacking-Enet stacking ensemble model exhibits three main advantages. First, our model was validated in two large-scale independent cohorts, ensuring its generalization ability. Munquad et al. developed a biologically interpretable deep learning framework based on a convolutional deep neural network (CDNN) for subtype classification (Munquad et al., 2022). Their model integrated transcriptome and methylome to accurately predict molecular subtypes. However, their model was trained and validated on a single-center cohort and lacked external validation. Second, the XGBoost.Enet-stacking-Enet model exhibits better interpretability, aiding us to better understand the decision-making process. Mao et al. developed a CDNN to classify samples (Mao et al., 2022). However, their models suffer from poor interpretability and limited clinical translation potential. Third, we developed a website tool to deploy machine learning models to make it easier for clinicians to access. Ensenyat-Mendez et al. developed GBM subtype classifiers based on transcriptomic and epigenomic using random forest and nearest shrunken centroid algorithms (Ensenyat-Mendez et al., 2021). Tang et al. leveraged extreme gradient boosting (XGBoost) to develop a classifier to predict the molecular subtypes of GBM (Tang et al., 2021). The two models mentioned above have not been deployed on web tools, making them inconvenient for clinicians to use.

The innovation of this model lies in two key points: 1) the source of its features and 2) the model stacking strategy. Our features were obtained from eccDNA molecular profiling. We first revealed the underlying correlation between eccDNA and the molecular subtypes of IDH wild-type GBM. A stacking ensemble strategy was developed based on enumeration. The ecGBMsub framework maximizes the advantages of stacking ensemble models by integrating diverse base models and meta-models to capture complex relationships within datasets. The framework enhances model stability, especially when dealing with challenging datasets such as imbalanced or high-dimensional data.

However, there are some limitations. First, the eccDNAs identified by Circle-seq sequencing need to be further experimentally verified to eliminate false positive effects. Likewise, there may be eccDNAs that are not detected by Circle-seq due to false negative effects. More advanced sequencing methods and computational biology tools are needed to fully corroborate and supplement our detected eccDNA molecules. Second, all data are sourced from public datasets, and a large-scale in-house cohort is needed to further verify the robustness of the model. Third, to confirm that eccDNA molecules play a role in subtype transformation, *in vitro* and *in vivo* experiments that target specific eccDNA molecules are needed. Finally, our models are trained and tested based on the transcription profile of the genes carried by eccDNA, which can be attributed to the lack of a large eccDNA molecular profile cohort. In the future, artificial intelligence models based on eccDNA’s DNA molecular profiling should be developed. Such models might be capable of monitoring the emergence and evolution of eccDNAs during disease progression to achieve much-needed patient stratification.

## Data Availability

The original contributions presented in the study are included in the article/Supplementary Material, further inquiries can be directed to the corresponding authors.
