# Supplementary figures and images for: ecGBMsub: an integrative stacking ensemble model framework based on eccDNA molecular profiling for improving IDH wild-type glioblastoma molecular subtype classification
Source: Front Pharmacol. 2024 Apr 11;15:1375112. doi: 10.3389/fphar.2024.1375112 (PMC11043526; doi:10.3389/fphar.2024.1375112)

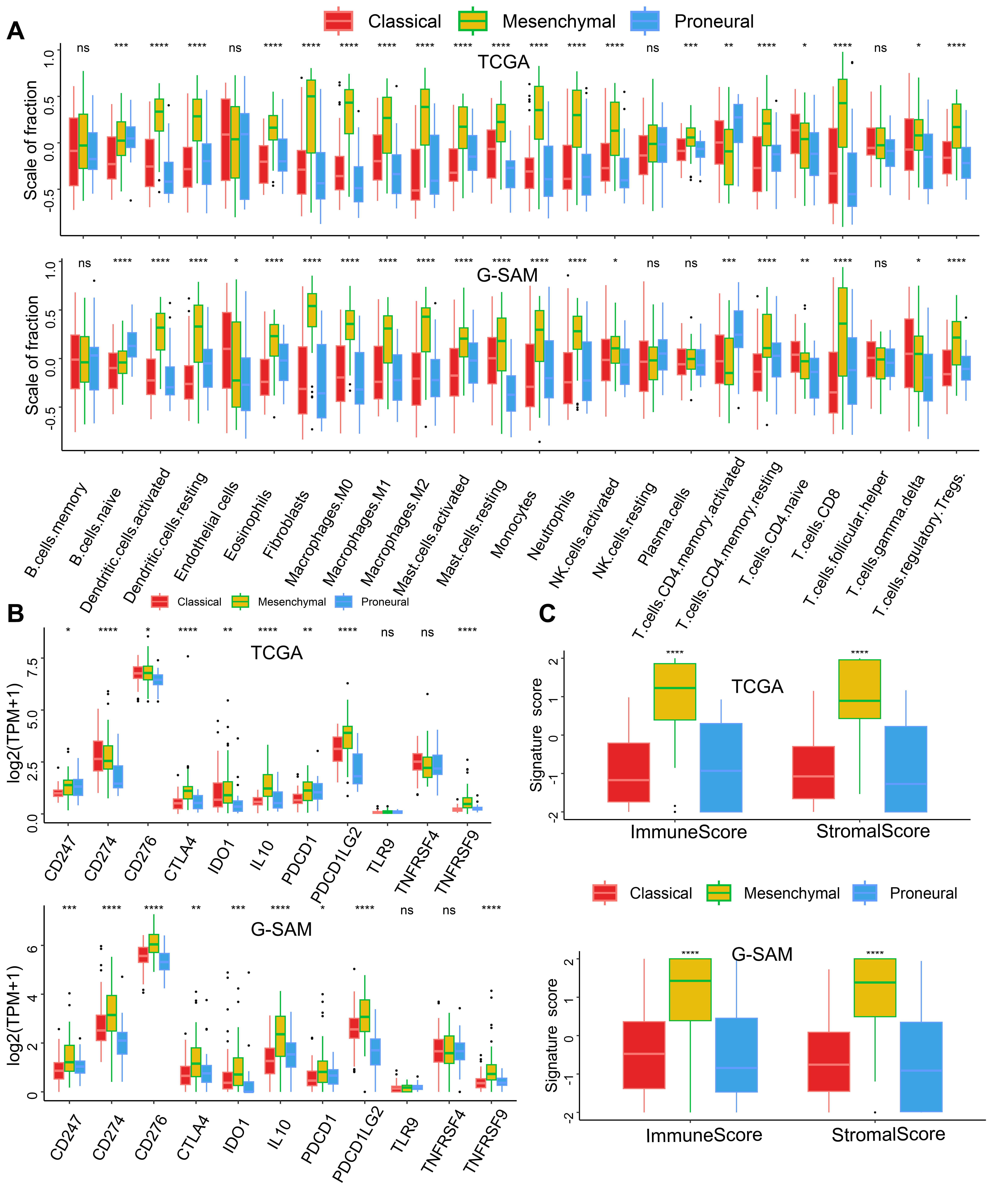

Supplement: Supplementary file 1 [file Image3.TIF]

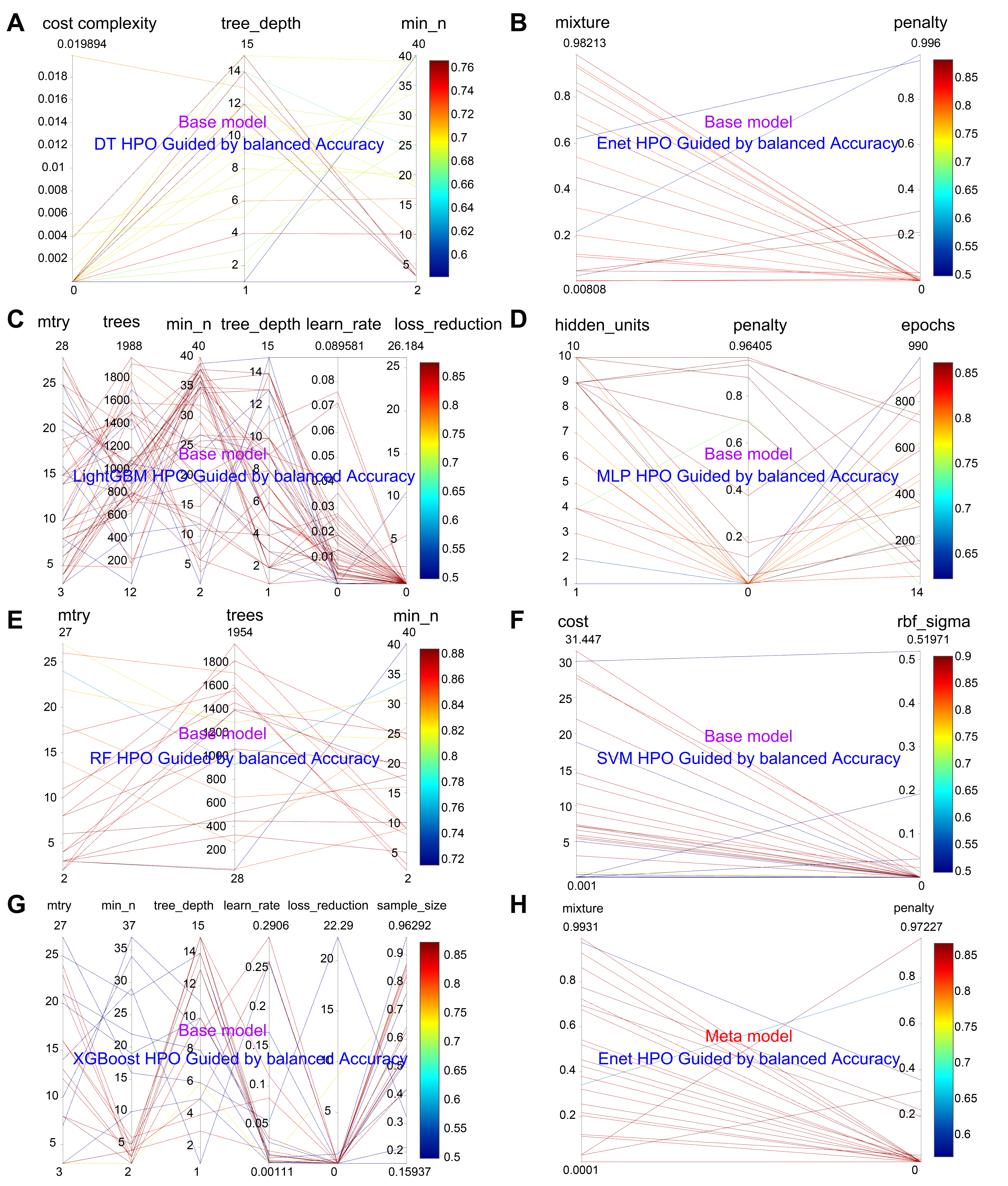

Supplement: Supplementary file 2 [file Image2.TIF]

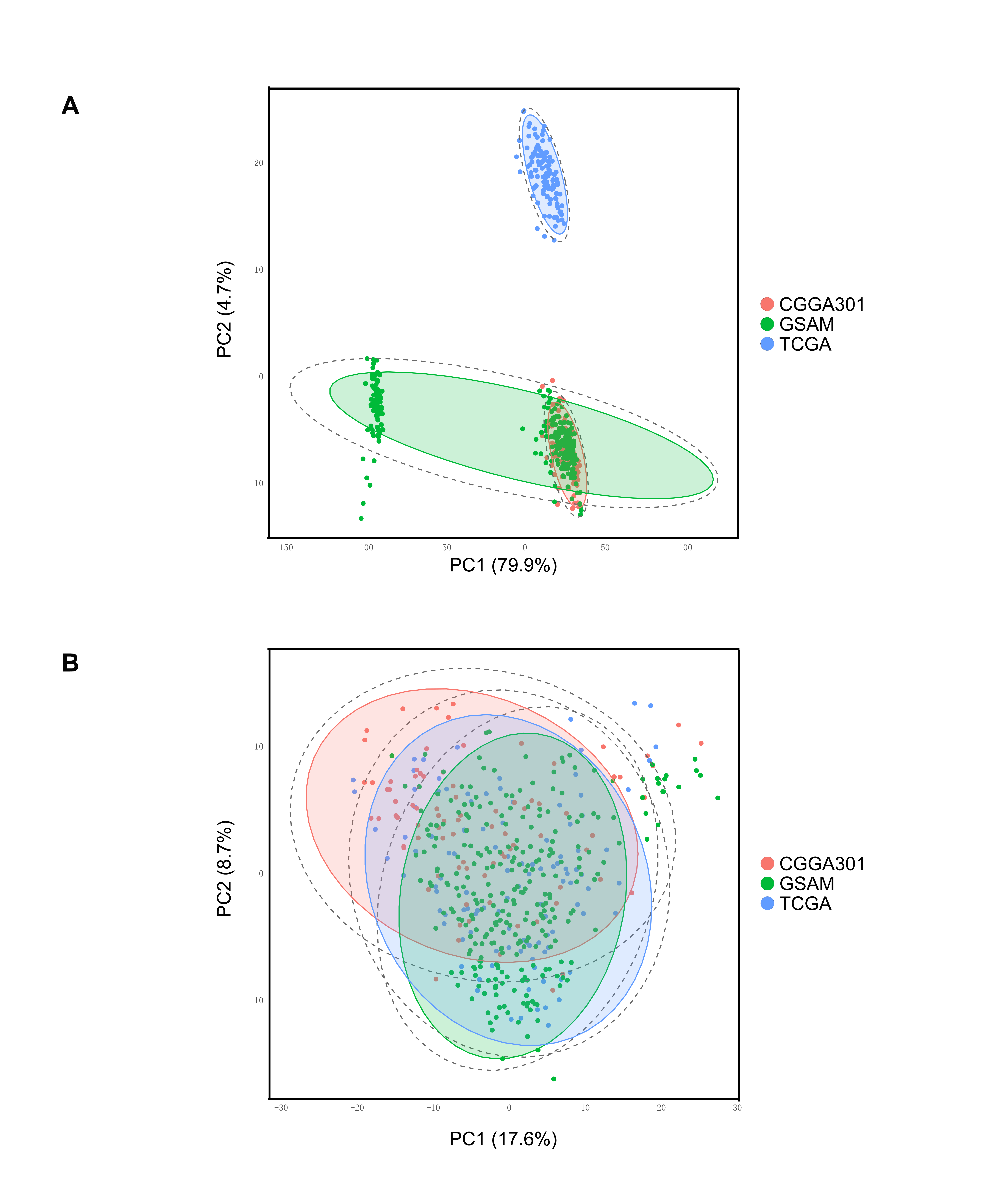

Supplement: Supplementary file 3 [file Image1.TIF]
